# Self Perceived Emotional Functioning of Spanish Patients with Amyotrophic Lateral Sclerosis: A Longitudinal Study

**DOI:** 10.3389/fpsyg.2012.00609

**Published:** 2013-01-08

**Authors:** Jesús S. Mora, Teresa Salas, María L. Fajardo, Lourdes Iváñez, Francisco Rodríguez-Santos

**Affiliations:** ^1^ALS Unit, Department of Neurology, Hospital Carlos IIIMadrid, Spain; ^2^Department of Epidemiology, Consejería de Salud de AndalucíaHuelva, Spain; ^3^Department of Epidemiology, Consejería de Salud de AndalucíaSevilla, Spain; ^4^Department of Clinical Psychology, School of Psychology, Universidad AutónomaMadrid, Spain

**Keywords:** amyotrophic lateral sclerosis, ALS, quality of life, emotional functioning, ALSAQ-40, self evaluation, depression, hopelessness

## Abstract

**Background:** ALS is a neurodegenerative disease of the entire motor system that most frequently ends with respiratory arrest in few years. Its diagnosis and the rapid progression of the motor dysfunctions produce a continued emotional impact. Studies on this impact are helpful to plan adequate psychotherapeutic strategies. **Objective:** To assess and analyze: First: How the patients with ALS perceive their emotional health. Second: The emotional impact of their physical disabilities. Third: The physical disabilities with highest emotional impact. Fourth: The feelings with highest emotional impact. **Methods:** Up to 110 Spanish patients with ALS were assessed less than 1 year from diagnosis, then twice more at 6 month intervals, using the ALS Quality of Life Assessment Questionnaire (ALSAQ-40) validated for use in Spanish. Descriptive analysis and correlation between variables were obtained. **Results:** Worries about the future, of lack of freedom, and of being a burden were prevalent feelings. On average depression was felt only “sometimes.” Only 25% of the variations in the emotional state were explained by changes in the physical state at first evaluation, and 16% at the last one. Emotional functioning correlated significantly with the physical disabilities at first and second evaluation, less so at third. Communication disabilities always had the highest impact. Depression at first evaluation and hopelessness at the next two evaluations had the highest emotional impact. Hopelessness did not correlate with any physical disability at the third evaluation. On the whole, emotional dysfunction was self perceived as intermediate (between none and worst), and remained stable at 1 year follow up, in both bulbar and spinal onset patients. **Conclusions:** Physical dysfunctions *per se* have a limited role in patients´ emotional distress. Communication disabilities, as well as feelings of depression at early stages of illness, and of hopelessness later on, had the most impact. This requires their careful therapeutic attention. On average, Spanish patients with ALS cope with their disease, overcoming depression, which is not felt often, and with just mid levels of emotional dysfunction.

## Introduction

ALS causes a progressive and generally rapid degeneration of the entire motor neuron system and, as a result of the subsequent muscle denervation, atrophy and weakness of the skeletal muscles, including limbs, bulbar, and respiratory muscles. Mean survival is about 3.5 years. 50% of patients may survive 3 years, 20% 5 years, and less than 10% 10 years. Minor cognitive deficits have been documented in up to 50% of patients, but 5–13% of patients may also develop frontotemporal dementia (FTD). ALS and FTD may have genetic overlapping (Mora Pardina, 2011). At present, ALS does not have a cure, or even a treatment that patients might perceive as clinically significant. Patients experience the progression of their disease as their physical disabilities progress and extend relentless. People suffering from ALS go through a tremendous amount of continuous emotional distress, right after a shocking diagnosis. The rapid decline in skeletal muscle strength causes physical dependence up to for the basic activities of daily life (ADL), which entails complex and mixed emotional reactions. The disease affects not only the patient but also their caregivers and family members, all of whom face hard emotional challenges that interrelate with those of the patient (Salas and Lacasta, 1999; Pagnini et al., 2010a,b).

We often observe that severe physical deficits do not systematically correlate with increased levels of depression, self perception of poor quality of life (QoL), or high emotional distress (Salas and Lacasta, 1999), as others have also observed (Pagnini, 2012). Moreover, the impact the disease has on emotional well-being differs significantly between individuals, and the psychological reaction of the patients to their disease and their ability to cope has an effect on the disease’s evolution (Chiò et al., 2004; Roach et al., 2009; Montel et al., 2012). Therefore, the traditional assumption of a tight correlation between rapidly progressive severe physical disabilities and emotional distress ought to be reconsidered. To study the relationship between the different physical disabilities caused by the disease and the emotional impact they may produce for the patient may contribute to better understanding and treatment of the emotional distress that ALS patients and their families endure. We understand that the way patients cope with their disease may be influenced by their cultural background; this is the first study to look at a Spanish population.

### Objective

To assess and analyze, at different times during their disease:

First: How the patients with ALS perceive their own emotional health.
Second: The emotional impact of each one of their physical disabilities.
Third: The physical disabilities with the highest emotional impact.
Fourth: The feelings with the highest emotional impact.

## Materials and Methods

### Measuring tool

We considered the ALS Assessment Questionnaire (ALSAQ-40) to be the most appropriate measuring tool for our objectives. The ALSAQ-40 is a specific state of health self appraisal questionnaire for ALS patients that looks at physical and emotional areas considered important by patients (Jenkinson et al., 1999; Epton et al., 2009). The ALSQ-40 has been validated for use in the Spanish language (Salas et al., 2008).

The ALSAQ-40 consists of 40 questions assessing four physical and one emotional area or dimension as self perceived by the patient. These are Physical Mobility (MOB), Independence/ADL (ADL), Eating and Drinking (EAT), Communication (COM), and Emotional Functioning (EMO). The first four dimensions assess physical deficiencies or disabilities that occur along with the disease. The fifth area evaluates the way that the patient deals emotionally with the disease. Each question or item is scored 0 to 4 according to the frequency of the symptom or feeling (never, rarely, sometimes, frequently, always). From the direct scores it is possible to obtain for each dimension an index ranging from 0 (best state of health) to 100 (worst state) which allows comparisons to be made between dimensions or over time. This ALS specific QoL measuring tool has demonstrated high validity, consistency, and reliability and appears sensitive to changes that have an impact on the overall status of patients (Jenkinson et al., 1999; Epton et al., 2009). It has been validated in different languages (Yamaguchi et al., 2004; Maessen et al., 2007; Salas et al., 2008; Palmieri et al., 2010; Pavan et al., 2010).

### Patient sample and methods

A total of 231 Spanish ALS patients were originally included in a 4-year long study. All were diagnosed with probable or definite ALS according to the El Escorial diagnostic criteria. None had clinical evidence of FTD, and all were receiving treatment with riluzol, the only approved ALS drug. They were living in different regions of Spain but they attended the ALS clinic at differing intervals. Of the total, only 110 patients whose first assessment was done no later than 12 months after diagnosis have been included in this study, in order to ensure a homogenous sample in terms of disease timeline. The evaluation of patients was longitudinal. Each patient filled out the questionnaire at three different times at 6 month intervals. Responses were always written or dictated by the patient. The questionnaire was first mailed accompanied by a request and an explanatory letter, and it was returned in clinic or by mail. The second and third evaluations were done in clinic or at home if the patient could not attend the clinic, and then mailed to us. The questionnaire could be completed in 10 min.

### Statistical analysis

Independent or explanatory variables were age, gender, site of onset (bulbar vs. spinal), time from onset of symptoms, time from diagnosis, and 30 functional variables, questions or items taken from the physical areas of the ALSAQ-40: Items 1–10 (MOB), 11–20 (ADL), 21–23 (EAT), and 24–30 (COM). Dependent variables were the 10 items, 31–40 (EMO) from the emotional dimension. These are:

1. I have felt lonely
2. I have been bored
3. I have felt embarrassed in social situations
4. I have felt hopeless about the future
5. I have worried that I am a burden to other people
6. I have wondered why I keep going
7. I have felt angry because of the disease
8. I have felt depressed
9. I worried about how the disease will affect me in the future
10. I have felt as if I have no freedom

The statistical analyses were descriptive, of correlation (regression analysis, Pearson’s correlation), and of mean differences (*t*-test for independent samples), using SSPS v.15.0 for Windows, Microsoft Excel, and EPI INFO 2000.

## Results

### Demography

The sample size was 110 patients, 50 women and 60 men. 70 patients had spinal onset, 40 had bulbar onset (Table 1). Mean age at the first evaluation of 59 ± 14 years. Mean time between diagnosis and first assessment was 5.8 ± 4.0 months. The second evaluation was completed 5.7 ± 1.5 months after the first one by 76 patients (69.1%). The third assessment was filled out 6.5 ± 1.0 months later by 52 patients (47.3%).

**Table 1:** **Sample distribution by gender and site of onset**.

	Bulbar	Spinal	Total
Women	24 (21.8%)	26 (23.7%)	50 (45.5%)
Men	16 (14.6%)	44 (39.9%)	60 (54.5%)
Total	40 (36.4%)	70 (63.6%)	110 (100%)

### Self perception of emotional health

**Figure 1:**
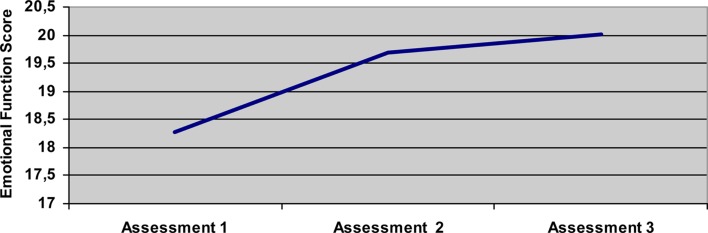
**Evolution of EMO along the three evaluations for the total sample**.

In spite of increasing scores in the physical areas (MOB, ADL, EAT, COM) as the disease progresses, the emotional area (EMO) scores did not significantly increase (*t*-test for independent samples; Table 2). During the three evaluations, there are no significant differences in EMO between patients with bulbar and spinal onset, in spite of them having significant differences in physical functions: higher MOB scores in spinal onset patients, and higher EAT and COM scores in bulbar onset patients, as expected (Table 2). During the study period, mean EMO scores remain at around mid level, between best and worst. There are no significant differences in EMO scores between the first and third evaluations (1.74 points), and even less between the second and third evaluations (0.33 points; Figure 1).

**Table 2:** **Mean and SD for each area in bulbar and spinal onset**.

	MOB		ADL		EAT		COM		EMO	
	Bulbar	Spinal	Bulbar	Spinal	Bulbar	Spinal	Bulbar	Spinal	Bulbar	Spinal
Maximun score	40	40	40	40	12	12	28	28	40	40
First evaluation	14.5 ± 11.4	19.8 ± 9.7	18.1 ± 12.9	20.6 ± 11.6	8.1 ± 3.6	1.9 ± 2.9	20.6 ± 7.5	4.6 ± 6.1	19.7 ± 13.1	17.4 ± 8.4
Second evaluation	19.3 ± 13.1	25.1 ± 11.9	23.9 ± 13.3	25.4 ± 11.6	9.4 ± 3.4	2.2 ± 3.5	23.5 ± 6.5	8.8 ± 9.3	21.0 ± 9.7	19.1 ± 10.5
Third evaluation	21.6 ± 12.9	29.4 ± 10.9	24.9 ± 12.7	30.7 ± 11.3	9.6 ± 2.4	4.3 ± 4.4	23.0 ± 5.6	11.6 ± 10.2	19.3 ± 9.4	20.3 ± 11.7

At the first evaluation, less than 12 months after diagnosis, the feelings most often felt (that is, a score higher than 2: “*sometimes*”) were worries about how the disease will affect the patient in the future (2.77), and feelings of being a burden to others (2.45), of lack of freedom (2.24), and of hopelessness (2.21). Anger at the disease (2.00) and depression (1.94) came after, but scored higher in patients who had been diagnosed less than 6 months ago (Table 3). At the second evaluation, about 6 months later, the feelings most often felt remain the same: worries about the future (2.66), lack of freedom (2.64), being a burden (2.53), hopelessness (2.33), and anger (2.22). Depression remained lower than 2 (1.91). At the third evaluation, 1 year after the first one, feelings of lack of freedom (2.98), worries about the future (2.73), and of being a burden (2.60) got close to a score of 3: “*frequently*.” Hopelessness (2.21), anger (2.06), and depression (2.02) maintained similar scores to the first evaluation (Table 3).

**Table 3:** **Mean scores and SD of EMO items at the three evaluations**.

EMO items*	First evaluation	Second evaluation	Third evaluation
1. I have felt lonely	0.98 ± 1.3	1.16 ± 1.5	1.10 ± 1.5
2. I have felt bored	1.30 ± 1.3	1.64 ± 1.5	1.67 ± 1.4
3. I have felt embarrassed in social situations	1.07 ± 1.3	1.10 ± 1.4	1.15 ± 1.3
4. I have felt hopelessness about the future	2.21 ± 1.2	2.33 ± 1.4	2.21 ± 1.5
5. I have worried about being a burden to other people	**2.45 ± 1.2**	**2.53 ± 1.2**	**2.60** **± 1.4**
6. I have wondered why I keep going	1.30 ± 1.4	1.49 ± 1.5	1.56 ± 1.5
7. I have felt angry because of the disease	2.00 ± 1.4	2.22 ± 1.4	2.06 ± 1.5
8. I have felt depressed	1.94 ± 1.2	1.91 ± 1.3	2.02 ± 1.4
9. I have worried about how the disease will affect me in the future	**2.77 ± 1.0**	**2.66 ± 1.3**	**2.73** **± 1.4**
10. I have felt as if I have no freedom	**2.24 ± 1.4**	**2.64 ± 1.5**	**2.98** **± 1.3**

*Items are scored as: Never = 0, Rarely = 1, Some times = 2, Frequently = 3, Always = 4.

*Bold indicates higher values that are most significant*.

### Emotional impact of physical disabilities

**Figure 2:**
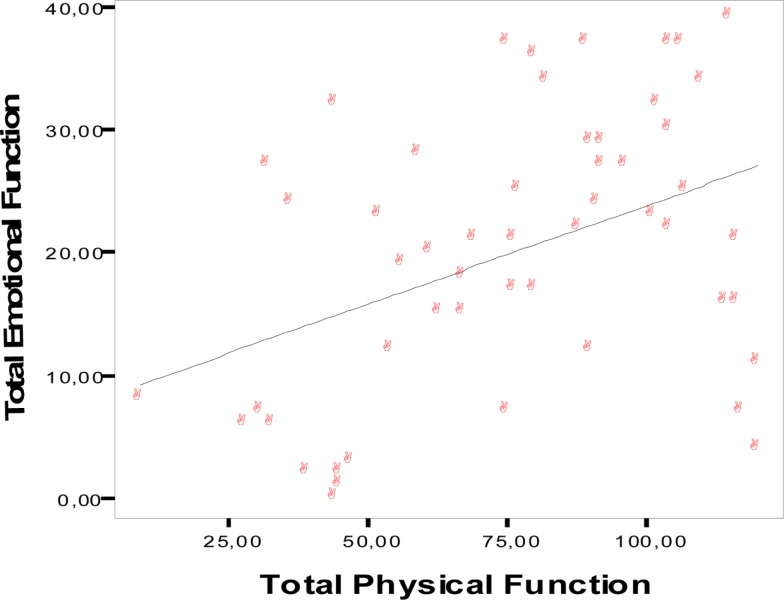
**Dispersion graph for emotional functioning and total combined physical functions at the third evaluation**.

To calculate how much the physical disability may be directly responsible for the emotional distress experienced by the patients, we carried out a dispersion diagram and calculated its linear regression. We used the EMO score as dependent variable and the sum of the scores of the physical areas (MOB, ADL, EAT, COM) as independent variables. The null hypothesis establishes that there is no relationship whatsoever between the two variables. To calculate the linear regression with 5% error and 95% confidence interval, we obtained *y* = 8.298 + 0.191x with a correlation coefficient *R* = 0.51 and a determination coefficient of *R*^2^ = 0.25. This indicates that only 25% of the variations that occur in the emotional state are explained by the changes in the physical state. The same analysis at the last evaluation produced the linear regression equation *y* = 7.80 + 0.16x, with a correlation coefficient of *R* = 0.42 and a determination coefficient of *R*^2^ = 16. This shows an even smaller relationship between emotional and physical functions: only 16% of the variations that occur in the emotional state at the third evaluation, when patients experience more severe disabilities, are explained by the variations in the physical state (Figure 2).

### Physical disabilities with highest emotional impact

At the first evaluation there were significant correlations between EMO and COM, MOB and EAT (*p* < 0.01; bilateral, Pearson’s correlation) in that order, and less significantly with ADL (*p* < 0.05; Table 4). At the second assessment there were significant correlations with all four physical areas (*p* < 0.01), the highest seen with COM, then with ADL (Table 5). At the third evaluation, the highest correlation continued being with COM (*p* < 0.01), and less so with MOB and EAT (*p* < 0.05; Table 6).

**Table 4:** **Correlations between areas at the first evaluation**.

	ADL	EAT	COM	EMO
MOB	0.400**	0.001	−0.046	0.360**
ADL	1	0.095	0.017	0.242*
EAT		1	0.856**	0.307**
COM			1	0.398**

*p < 0.05 bilateral; **p < 0.01 bilateral.

**Table 5:** **Correlation between areas at the second evaluation**.

	ADL	EAT	COM	EMO
MOB	0.552**	0.022	0.165	0.331**
ADL		0.203	0.317**	0.346**
EAT		1	0.854**	0.342**
COM			1	0.444**

*p < 0.05 bilateral; **p < 0.01 bilateral.

**Table 6:** **Correlation between areas at the third evaluation**.

	ADL	EAT	COM	EMO
MOB	0.556**	0.278*	0.300*	0.330*
ADL	1	0.220	0.301*	0.267
EAT		1	0.845**	0.301*
COM			1	0.355**

*p < 0.05 bilateral; **p < 0.01 bilateral.

### Feelings with highest emotional impact

Since physical dysfunctions were only partially responsible for the emotional impact, with an impact ranging from 25% at first evaluation to 16% at last evaluation, as shown in section 2, we assessed correlations between the different emotional states in EMO and the total EMO. To assess which emotions are elicited by the different physical disabilities, we calculated correlations between each EMO item and MOB, ADL, EAT, and COM (Tables 7– 9). At the first evaluation all EMO items correlated highly with total EMO (*p* < 0.01 bilateral). The highest were items 8 on depression (Pearson *r* = 0.81), 7 on anger (*r* = 0.75), and 6 on keep going (*r* = 0.74). Item 8 on depression correlated highly (*p* < 0.01) with disabilities in MOB (*r* = 0.27) and COM (*r* = 2.5), and less so (*p* < 0.05) with ADL and EAT. All of them significantly less than with EMO (*r* = 0.81). Item 7 on anger correlated highly with MOB, COM, and ADL (*p* < 0.01) and less so with EAT (*p* < 0.05). Item 6 on keep going correlated highly with EAT, COM, and MOB. In all cases, the correlations were at much lower levels than with EMO (Table 7).

**Table 7:** **Correlations between EMO items and other areas at the first evaluation**.

EMO Items	MOB	ADL	EAT	COM	EMO
1. I have felt lonely	0.148	0.112	0.386**	0.399**	0.673**
2. I have felt bored	0.217*	0.183	0.249**	0.332**	0.625**
3. I have felt embarrassed in social situations	0.052	−0.011	0.433**	0.507**	0.540**
4. I have felt hopelessness about the future	0.294**	0.144	−0.049	0.017	0.670**
5. I have worried about being a burden to other people	0.265**	0.241*	0.087	0.133	0.702**
6. I have wondered why I keep going	0.274**	0.163	0.306**	0.290**	0.740**
7. I have felt angry because of the disease	0.297**	0.272**	0.227*	0.293**	0.751**
8. I have felt depressed	0.274**	0.214*	0.200*	0.254**	0.812**
9. I have worried about how the disease will affect me in the future	0.198*	0.154	0.054	0.154	0.712**
10. I have felt as if I have no freedom	0.441**	0.197*	0.168	0.309**	0.688**

*p < 0.05 bilateral; **p < 0.01 bilateral.

*Bold indicates higher values that are most significant*.

At the second assessment all EMO items kept a high correlation (*p* < 0.01) with total EMO. The feelings with highest correlations were items 4 on hopelessness (*r* = 0.85), 8 on depression (*r* = 0.84), and 10 on lack of freedom (*r* = 0.78). Item 4 on hopelessness and 8 on depression correlated highly with COM (*r* = 0.32) and less so with the other physical areas. Item 10 on lack of freedom correlated highly with COM, MOB, and ADL, and less so with EAT. All EMO items correlated highly with COM, except items 5 on being a burden and 9 on worries about future (Table 8).

**Table 8:** **Correlations between EMO items and other areas at the second evaluation**.

EMO Items	MOB	ADL	EAT	COM	EMO
1. I have felt lonely	0.081	0.106	0.416**	0.462**	0.620**
2. I have felt bored	0.253*	0.406**	0.351**	0.408**	0.757**
3. I have felt embarrassed in social situations	0.170	0.125	0.337**	0.371**	0.561**
4. I have felt hopelessness about the future	0.271*	0.260*	0.241*	0.324**	0.847**
5. I have worried about being a burden to other people	0.216	0.289*	0.085	0.123	0.704**
6. I have wondered why I keep going	0.290*	0.252*	0.235*	0.295**	0.766**
7. I have felt angry because of the disease	0.290*	0.295**	0.223	0.334**	0.769**
8. I have felt depressed	0.254*	0.252*	0.181	0.326**	0.844**
9. I have worried about how the disease will affect me in the future	0.088	0.066	0.118	0.162	0.658**
10. I have felt as if I have no freedom	0.477**	0.450**	0.256*	0.380**	0.783**

**p < 0.01 bilateral; *p < 0.05 bilateral.

*Bold indicates higher values that are most significant*.

At the third evaluation every EMO item maintained its high correlation with total EMO. The feelings with the highest correlations were items 4 on hopelessness (*r* = 0.91), 6 on keep going (*r* = 0.84), 2 on feeling bored (*r* = 0.83), and 8 on depression (*r* = 0.80). Item 4 on hopelessness and item 6 on keep going did not correlate with any of the physical disabilities. Item 2 on boredom correlated highly with EAT and less so with MOB and COM. Item 8 on depression correlated highly with COM and MOB and less so with ADL. Item 10 on lack of freedom was highly correlated (*p* < 0.01) with the five areas (Table 9).

**Table 9:** **Correlations between EM items and other areas at the third evaluation**.

EMO Items	MOB	ADL	EAT	COM	EM
1. I have felt lonely	0.254	0.011	0.149	0.145	0.682**
2. I have felt bored	0.319*	0.163	0.364**	0.298*	0.826**
3. I have felt embarrassed in social situations	0.164	0.174	0.255	0.317*	0.634**
4. I have felt hopelessness about the future	0.238	0.220	0.076	0.152	0.913**
5. I have worried about being a burden to other people	0.234	0.286*	0.138	0.219	0.698**
6. I have wondered why I keep going	0.193	0.166	0.143	0.209	0.836**
7. I have felt angry because of the disease	0.233	0.181	0.245	0.152	0.777**
8. I have felt depressed	0.370**	0.313*	0.339*	0.494**	0.801**
9. I have worried about how the disease will affect me in the future	0.121	0.186	0.205	0.299*	0.762**
10. I have felt as if I have no freedom	0.425**	0.377**	0.433**	0.488**	0.764**

**p < 0.01 bilateral; *p < 0.05 bilateral.

*Bold indicates higher values that are most significant*.

## Discussion

This study shows that as the physical deterioration of the patients with ALS increased, the self perception of the emotional distress remained more or less stable, and this happened in both spinal and bulbar onset patients, who have different timings of their physical disabilities. Emotional distress was felt, on average, as intermediate between none or worst. At the first evaluation the most frequent worries, felt more than *sometimes* but less than *frequently*, were those about the future, of losing autonomy, of being a burden, and then of despair. Less frequently felt, just *sometimes*, were depression and anger, which were more frequently felt nearer the time of diagnosis. This pattern continued at the second evaluation, with a slight increase in loss of freedom and with depression felt slightly less than *sometimes*. At the last evaluation, between 18 and 24 months after diagnosis, the pattern continues but lack of autonomy is the prevalent feeling followed by fear of the future and of being a burden, these three feelings got closer to being felt *frequently*. Depression remained stable, just being felt *sometimes*.

This pattern suggests that, for Spanish patients attending an ALS unit, the most frequent distressful thoughts are those related to fear of the future and of loss of autonomy with concerns for loved ones. Feelings of hopelessness, of depression and anger were felt less often. This might be explained by the attendance of a specialized clinic whose professionals try to fight those negative feelings with constant information on research, clinical trials, and psychological surveillance. Other explanations could be linked to the patients´ shared cultural and social background.

All four physical disabilities correlate with EMO, but it is the COM disability that has the most emotional impact as soon as it appears. This implies that dedicated care of this disability is important since it is a cause of significant distress. On the whole, physical disabilities accounted for only 25–16% of the emotional distress, and less so as the disease advances. Other varied psychosocial factors may contribute to modify the EMO (Pagnini, 2012). However, each negative feeling tested correlated highly with the global emotional dysfunction. It seems plausible that any psychosocial factor, such as advanced health care, strong familial, and social support, spirituality, or an optimistic personality, could influence the EMO of the patient by changing their patterns of feelings or emotions. Negative thoughts, not the physical disabilities *per se*, may account for an important part of the emotional distress felt by patients. We have known for a long time that emotional distress does not depend on the physical damage that one experiences but on the way that the mind processes the experience (adapted from Epicurus, 300 B.C.: “*To humans, the things do not affect us by themselves, but by the way we interpret them*”).

All 10 feelings or emotional states tested correlate strongly with the global EMO, but each one has a different weight as representative of the patient’s EMO at different times. Less than 1 year after diagnosis depression is the feeling with most emotional impact, followed by anger, although on average they were felt only “*some times*.” Both are highly related to the dysfunctions in COM and MOB. Once most disabilities are present, 18 months after diagnosis, feelings of hopelessness reach the same power as depression, with the COM disabilities contributing most to these feelings. Two years after diagnosis, most feelings have increased their impact, but those of hopelessness and of stopping fighting are the most representative. Worries about the future and of lack of freedom are perceived often but produce a lesser impact. Interestingly, except for lack of freedom, none of the emotional states are related to the severity of the physical dysfunctions at this stage of the disease. In this way, they are negative states of mind, not directly related to the physical disabilities, and so, subject to be treated by dedicated psychotherapy.

Many studies have tried to determine what is called the QoL of patients with ALS, and many scales have been created that try to measure it. QoL may be a confusing concept in this dreadful disease, which makes it more difficult to “measure.” The methodological limitations inherent to this measurement contribute to a wide range of reported results, and statements on QoL must be considered dependent of the scale used. An excellent review has recently been published (Pagnini, 2012). QoL has been reported to be negatively correlated to suffering, a sense of burden, and hopelessness, and positively correlated to social support (Epton et al., 2009). Its relation with physical functioning was unclear to some (Ganzini et al., 1999) and it was more related to psychosocial rather than physical aspects of life, particularly in those patients with physical disabilities (Clarke et al., 2001). QoL, as assessed by the patient, has not been correlated to physical function or strength, but to psychological and existential factors, as well as to spiritual factors and support systems (Simmons et al., 2000, 2006). Hopelessness in ALS has been correlated with suffering and negatively with QoL (Ganzini et al., 1999). Hope did not correlate with physical and respiratory functions (Fanos et al., 2008). The real prevalence of depressive and anxious symptom in ALS is unclear (Norris et al., 2010; Taylor et al., 2010) although some studies found depressive symptoms common, and severe in 11–15% of the patients (McLeod and Clarck, 2007). Swallowing and breathing difficulties have been related to depressive symptoms, however the severity of the disease as such or its duration were not (Hillemaker et al., 2004). Most of these studies are in accordance with our findings.

An evaluation of 28 studies concluded that clinically significant depression is neither as prevalent nor as severe as might be expected and that patients are more likely to present with hopelessness and end-of-life concerns than clinically significant depression (Averill et al., 2007), this is consistent with our findings. Thirty ALS patients showed good psychosocial adjustment and subjective QoL, mild depressive symptoms, and no clinically relevant depression (Lulé et al., 2012). Another study rated QoL on average as satisfactory and found a moderate positive relation to physical impairment and a weak negative relation to time since diagnosis (Kubler et al., 2005). Despite marked deterioration in the patients’ health, there was no change in mental well-being and QoL. Psychological well-being appeared more important in maintaining QoL than physical factors (O’Doherty et al., 2010). Few changes were observed in the QoL of 35 patients over time (Olsson et al., 2010). The general QoL changed little overtime in 60 patients (Robbins et al., 2001). Individual QoL remained stable in 42 patients and was not dependant on physical function but on family, friends/social life, health, and profession (Neudert et al., 2004). There was substantial steadiness of QoL in 31 patients over 9 months (Gauthier et al., 2007). No relationship between EMO and muscle strength or functional ability was found in 30 patients (Palmieri et al., 2010). Most of these studies are in accordance with our findings.

What emerges from our study and others is that for ALS patients, as a group, physical dysfunction *per se* is not the main contributor to emotional suffering. Emotional suffering seems more related to negative feelings linked to the new physical condition. However, emotional dysfunction, and depression at its paradigm, do not seem to be exaggerated in spite of severe physical dysfunction and remain somehow stable along most of the disease. Terminal phases need additional studies. Since we do not agree with the idea of ALS affecting only nice and suffering-resilient people, we understand that ALS patients as a group behave as any other human group who try to cope with misfortune the best they can. As in any human disaster the emotional health of each ALS patient is not completely dependent on his/her physical strength but on the strength of the coping mechanisms that he/she may build to deal with the situation These mechanisms may be individual, familial, social, or spiritual, health care provided or other. It is difficult and risky to extrapolate results obtained from groups to individual patients since the individual variables that influence the specific QoL of each person are so varied. To facilitate the work of the therapists, our study tries to understand the contribution of specific negative feelings and worries, and their subsequent emotional states, to the emotional suffering of the patients throughout their disease, and which specific physical disabilities are most related to them. Specific psychotherapeutic interventions should be implemented to help the patient to overcome them. Development of adequate protocols is needed (Pagnini et al., 2012).

## Conflict of Interest Statement

The authors declare that the research was conducted in the absence of any commercial or financial relationships that could be construed as a potential conflict of interest.
